# Crossing the health misinformation crisis: Lessons from the giant hammerhead flatworm

**DOI:** 10.1590/0037-8682-0212-2025

**Published:** 2025-10-17

**Authors:** Hudson Alves Pinto, Alan Lane de Melo, Vitor Luís Tenório Mati

**Affiliations:** 1 Universidade Federal de Minas Gerais, Departamento de Parasitologia, Belo Horizonte, MG, Brasil.; 2 Universidade Federal de Lavras, Departamento de Medicina, Lavras, MG, Brasil.

**Keywords:** Giant hammerhead flatworm, Bipalium kewense, Health misinformation, Citizen science, Epistemology

## Abstract

We examined the recent surge in misinformation surrounding giant hammerhead flatworms (Bipaliinae, primarily *Bipalium kewense*), which has generated widespread confusion across multiple countries regarding their risks to human health. Prompted by a routine taxonomic identification, this study aims to restore scientific accuracy and address the growing disconnect between science and the public concerning these worms, which have become an Internet sensation. In late 2023, we were asked to identify specimens collected in Pedro Leopoldo, Minas Gerais, Brazil, that had already attracted considerable online and regional media attention, warning of a “new, invasive, and dangerous worm” and its supposed threats. After confirming the species was *B. kewense*, we reviewed the literature, constructed a timeline of scientific and media reports, and compared information retrieved from Google News and Google Scholar. Our findings indicate that while academic research on invasive species has steadily increased, sensationalized and often inaccurate content on hammerhead worms proliferated in the general media, particularly between 2017 and 2018. Notably, claims (2023-2024) regarding the toxicity of *Bipalium* spp. lack scientific support. This case illustrates the broader challenges of health misinformation in the digital age, where misleading narratives rapidly transcend countries and languages. We discuss potential drivers, including communication gaps between academia and the public, the influence of media and social networks, and behavioral factors underlying misinformation. Finally, we highlight the urgent need for innovative strategies and coordinated efforts to strengthen online science communication and ensure the dissemination of accurate information.

## INTRODUCTION

The deep-seated fear and disgust associated with repulsive and dangerous creatures-whether supposedly or genuinely deadly-are closely tied to human nature^1^ ^-^ ^3^. Evolutionary psychology employs the predator-defense model, or the concept of prepared learning, to explain fear of certain animals, such as snakes, which directly threatened the survival of our ancestors due to their venomous properties^3^ ^,^ ^4^. In contrast, the disease-avoidance model, which relates to the potential for disease transmission by various animals^1^ ^,^ ^3^ ^,^ ^5^, better explains psychological disorders such as fears and phobias associated with nonvenomous yet repulsive creatures, including worms^1^ ^,^ ^2^.

Aversion-inducing animals have long captured the attention of our hominid ancestors and, more recently in evolutionary terms, have permeated cultural consciousness since the earliest stages of human civilization. Health-related information, particularly concerning repulsive or poisonous animals, has traditionally been transmitted orally across generations^6^ ^,^ ^7^. Such knowledge was passed down through stories and cultural teachings, in which elders warned about dangers, and through rumors-an ancient practice that remains widespread today, especially on the Internet^8^ ^,^ ^9^. With the advent of written traditions, literate individuals became the primary keepers of knowledge. Since the Scientific Revolution, university scholars served as the principal conveyors of scientific understanding, sharing discoveries within relatively restricted academic circles. By the late 19th and early 20th centuries, newspapers and magazines-and later radio and television-began bringing science to a wider audience, underscoring the persistent challenges of effectively communicating knowledge through mass media^9^ ^,^ ^10^.

In the digital age, unrestricted Internet access and abundant information sources, including vast repositories of scientific journals, could be expected to improve public understanding of ancient emotional responses to worms. Paradoxically, however, this information overload has contributed to the proliferation of misinformation and fake news^8^ ^,^ ^9^ ^,^ ^11^ ^,^ ^12^. Against this backdrop, a Kafkaesque, yet real story has unfolded: the case of giant hammerhead flatworms (planarian species of the subfamily Bipaliinae) and the spread of health misinformation. This saga has spanned multiple countries, involved a diverse cast of actors, and inadvertently included the authors themselves. Considerable confusion has emerged worldwide regarding these unusual animals, extending into organizations and media far beyond academia, unlike in the past, when the subject remained largely confined to scholarly circles until the early 2010s. This mini-review, grounded in a transdisciplinary case study of a worm that has become an Internet sensation, highlights the disconnect between scientific knowledge and the broader community. We provide a comprehensive contextual overview, incorporate original metadata, and examine the significance of hammerhead flatworms for human and animal health. Our analysis spans their scientific characterization and fundamental biology, while extending to information science and epistemology, thereby offering a multifaceted perspective on this complex issue.

## PROFILE: HAMMERHEAD FLATWORMS

Although this case study did not primarily focus on zoological aspects, it began with a straightforward taxonomic diagnosis accessible to any professional with basic zoological training. In the second half of 2023, the discovery of vermiform organisms in Pedro Leopoldo (9°37′59.99″ S; 44°02′60.00″ W), a municipality in the metropolitan region of Belo Horizonte, Minas Gerais, Brazil, drew significant attention on the Internet and in regional news outlets (TV and radio). Reports warned of the emergence of a “terrible giant hammerhead worm”-a land planarian of the subfamily Bipaliinae named for the distinctive shape of its anterior end. The presence of this purportedly toxic organism near human habitations was quickly linked to risks for both humans and animals, as well as potential environmental impacts. Local journalists and concerned authorities reached out to us for information and guidance, and on November 1, 2023, three specimens of these flatworms were submitted for analysis.

**FIGURE 1:**
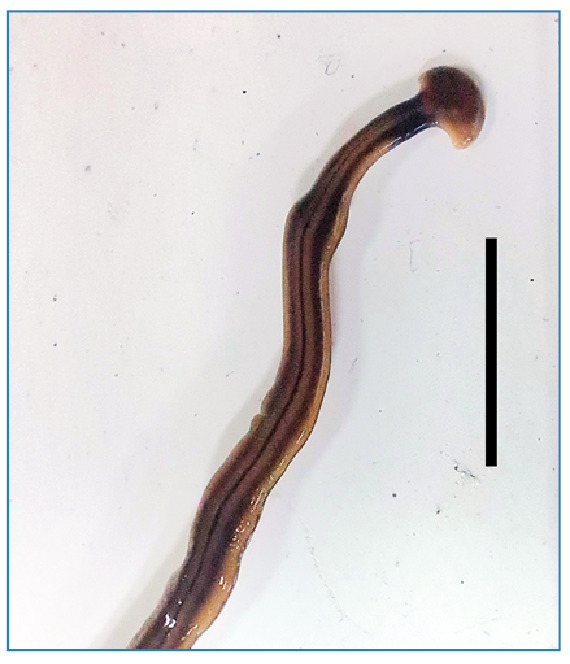
Specimen of *Bipalium kewense* collected in Minas Gerais, Brazil, in October 2023. The image shows the general morphology of the organism, highlighting the characteristic anterior region with an incomplet black collar, and longitudinal bands, which are distinctive traits of this species. Scale bar: 1 cm.

Specimens were examined morphologically under a stereomicroscope and identified, following Luna et al.^13^, as *Bipalium kewense* Moseley, 1878 (family Geoplanidae)^14^ ^-^ ^17^ (Figure 1). This invasive land planarian, native to Southeast Asia, now has a cosmopolitan distribution largely facilitated by human activities and is commonly observed in anthropogenically disturbed environments, such as gardens, greenhouses, and nurseries^17^ ^-^ ^20^. In Brazil, records of *B. kewense* date back to the late 19th century, with subsequent sightings documented across multiple regions of the country^21^ ^-^ ^24^, demonstrating that its presence is not a recent phenomenon.

For nearly two centuries, research on *Bipalium* spp. remained primarily an academic pursuit. However, in recent years, public attention and concern have increased, fueled by claims of ecological and health risks. After comparing information from online media sources with scientifically validated content from academic journals, additional questions emerged, prompting us to “follow the thread” of online discourse. This process led us to hypothesize that an Internet-driven bubble of misinformation and echo chambers had formed around this topic, with their origins and scope largely overlooked^8^ ^,^ ^9^. To test this hypothesis, we developed a methodological framework integrating media analysis with a review of the scientific literature. The following section details this approach to uncovering the dynamics of misinformation and its impact on public perception.

## METHODOLOGICAL PATHWAYS: TRACING THE FOOTPRINTS OF DIGITAL DECEPTION TO DECODE NARRATIVES

To investigate these events, we conducted a comprehensive literature review on hammerhead worms to construct a timeline of scientific and media accounts of these invertebrates. We systematically quantified and evaluated metadata, as well as the type, subject, and quality of online information over time. Reliability, authority, and breadth of evidence were assessed across both academic and non-academic sources. Parallel searches were performed by comparing scientific articles (including reviews and original research) from Google Scholar with media articles and news reports retrieved from Google News. To ensure comparability, identical search terms and timeframes (2005-2024) were applied. Initial search terms included the genus name *Bipalium* and the common names “hammerhead worm” and “hammerhead flatworm.” To isolate discussions on toxicity and environmental issues, searches were further filtered with the keywords “toxin” or “toxic,” or “poison” or “poisonous”, and “exotic”, “invasive” or “alien”. Each entry was reviewed individually. Records unrelated to *Bipalium* species, duplicates, and broken links were excluded. Google News entries were additionally categorized by tone using content analysis, with the following codes:

**Sensational/alarmist:** Fear or panic appeals; exaggerated or emotional language with alarmist terms; lack of a scientific basis.

**Informative:** Evidence-based explanations with references to reliable and verifiable sources; objective and educational language.

**Balanced/other:** Content neither clearly sensationalist nor purely informative; moderate, non-polarizing language; presentation of multiple perspectives with a balance between accurate information and emotional or engagement appeals.

All data were collected online and analyzed by one of the authors in January 2025.

## STATUS: FROM RISING ONLINE INTEREST TO THE SPREAD OF FAKE NEWS

**FIGURE 2:**
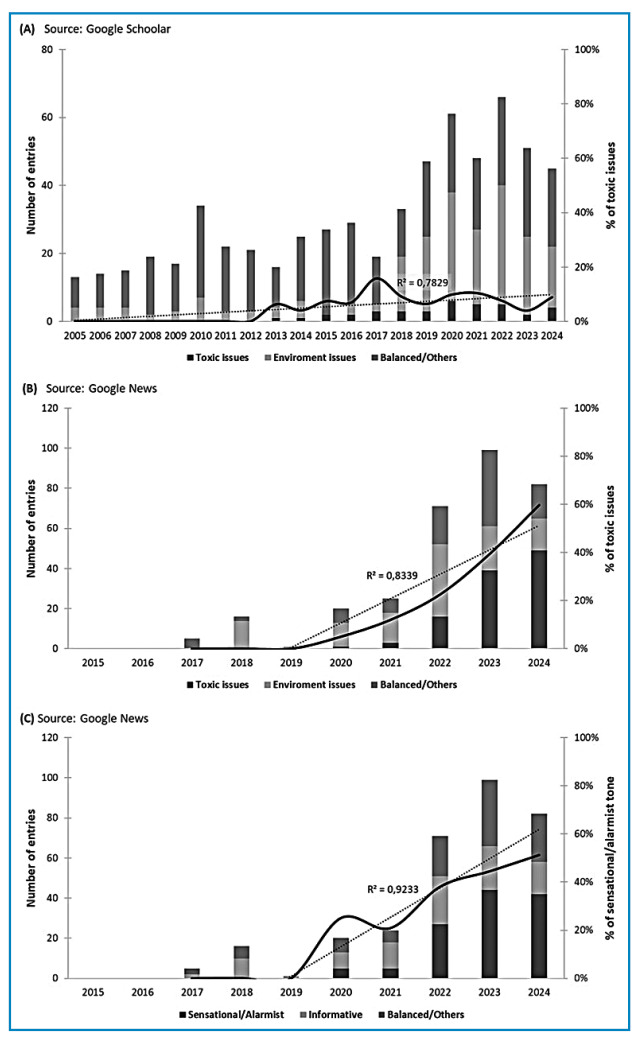
Temporal trends in scholarly and media coverage of hammerhead flatworms. Panels present raw counts (stacked bars) and percentages (lines) of main topic distributions in scientific articles indexed by Google Scholar **(A)** and in news articles, websites, and related sources indexed by Google News **(B)**. Topics are classified as toxic issues, environmental issues, or balanced/others across both datasets. Tone classification of Google News entries is shown in **(C)**, categorized as sensational/alarmist, informative, or balanced/others. Dashed trend lines for the percentages of toxic issues **(A,B)** and sensational/alarmist tone **(C)**, along with their respective coefficients of determination (R²), depict the strength of correlations over time.

The timeline of key scientific and media-related events with detailed findings is presented in Table 1 ^14^ ^-^ ^21^ ^,^ ^25^ ^-^ ^29^. Our qualitative and quantitative analyses show that, over recent decades, invasive species issues have remained the primary focus of researchers, with a modest rise in related publications (Figure 2A). This increase is proportionally lower than the overall surge in Google News entries (Figure 2B). Between 2016 and 2017, there was a marked rise in articles directed at audiences beyond the academic community. Initial public and media interest, particularly on Google News, centered on the invasion potential and environmental impact of these organisms. However, by 2023-2024, media coverage had shifted predominantly to the alleged toxicity of *Bipalium* spp., with frequent claims of risks to pets and humans through contact or ingestion of these planarians. These claims were unfounded. 

**TABLE 1:** Timeline of hammerhead flatworms worldwide, highlighting key scientific descriptions and discoveries, global spread, patterns of science-community engagement, impact types, and misinformation.

Year	Statement	Comments	Science-community engagement	Impact type
1831	First record.	Gray^14^ was the first to record hammerhead worms in Asia. The first described species, *Planaria lunata* (currently *Diversibipalium lunatum*), was named for the crescent-shaped anterior extremity.	Irrelevant*	N/A
1857	Genus description.	Stimpson^15^ described the genus *Bipalium* for terrestrial flatworms with broad, shovel-shaped heads collected during an expedition in the North Pacific. Today, it comprises over 160 species^18^ ^,^ ^19^.	Irrelevant	N/A
1874	Morpho-physiology.	Moseley^16^ described new *Bipalium* species, listed 16 others known in Southeast Asia, detailed their histology and comparative anatomy, and examined their behavior and carnivorous habits.	Irrelevant	N/A
1878	A new species.	*Bipalium kewense*, one of the largest hammerhead worm species, was described by Moseley from specimens already established in hothouses at the Royal Botanic Gardens, Kew, London^17^.	Irrelevant	N/A
1882	*B. kewense* in the Americas.	Collin (1882) and Sharp (1891) first reported the species in Brazil and the USA, respectively^21^ ^,^ ^25^. Subsequent records across the Americas appeared throughout the late 19th and 20th centuries^18^ ^-^ ^20^.	Irrelevant	N/A
1887	*B. kewense* in Europe.	Shortly after its initial description in England, its occurrence in continental Europe was reported, beginning with Germany^18^.	Irrelevant	N/A
1891	Earthworms in the diet.	Lehnert^26^ confirmed the carnivorous feeding habits of hammerhead worms, detailing how their pharynx envelops an entire earthworm segment.	Irrelevant	N/A
1900	Regenerative capacity.	The well-known totipotent regenerative capacity of planarians was studied and demonstrated in *Bipalium* ^27^.	Irrelevant	N/A
1983	*B. kewense* worldwide.	A century after its description, a literature review indicated that *B. kewense* was found on all continents except Antarctica, likely dispersed through the potted-plant trade from its native range (Vietnam to Cambodia)^18^.	Irrelevant	N/A
2009	First Internet news.	The first online news item on hammerhead worms described their appearance in Tuscaloosa, Alabama (USA), as “unusual” and “weird”, and claimed they destroyed European earthworm farms. An expert noted: “They are perfectly harmless to humans.”	Relevant	Positive
2014	TTX in *Bipalium*.	Stokes et al.^28^ reported tetrodotoxin (TTX) in *Bipalium* spp. in an open-access journal, suggesting its use in predation by paralyzing large prey via channel blockade.	Relevant	Positive
2016	Bioinvasive flatworms.	Sluys^20^ highlighted biodiversity risks posed by these globally invasive invertebrates, establishing a timeline of scientific developments and discussing control measures. Public awareness was raised with illustrations and guidelines.	Relevant	Positive
2017	Viral Facebook post.	A video and images of a hammerhead worm (*Bipalium* sp.) from Malaysia, posted on Facebook with the caption “What is this snake???,” went viral worldwide, garnering millions of views.	Major	Negative
2018	“Giant worms chez moi!”	A four-year citizen science survey in France, published in an open-access journal^29^, reported invasive *Bipalium* and *Diversibipalium* spp. The study mobilized blogs, Twitter, and media, while sensationalist news coverage popularized hyperbolic terms.	Important	Mixed**
2020	TTX in popular media.	A U.S. pop culture website published a sensationalist article on “snake-sized toxic worms”, claiming they produce the same neurotoxin as pufferfish. It warned against handling them without citing sources.	Important	Negative
2022	Hammerhead worms as pop icons.	Google News entries tripled (especially in the USA and Canada), dominated by sensationalist content. In contrast, scientific entries (Google Scholar) remained stable. Environmental topics prevailed in both datasets, but toxicity references surged in media.	Major	Negative
2023-present	“Likes” poison the truth!	Since 2023, toxicity has become a standard theme in non-scientific publications. Sensationalism now dominates coverage, leveraging unsupported claims that hammerhead worms threaten pets and humans. Scientists continue publishing on ecological issues, but authorities remain largely unresponsive.	Major	Negative

* Irrelevant: scientific information inaccessible to the general public. ** Mixed: media sensationalism altered positive scientific findings into dual outcome. N/A: Not applicable.

Stokes et al.^28^ identified tetrodotoxin (TTX) in these terrestrial invertebrates a decade ago, unintentionally providing material that has since been misused to fuel alarmist narratives in the 2020s. It is well established that ingestion of TTX from marine fish such as Japanese fugu (pufferfish) can cause poisoning, neurological complications, and even death^30^. However, the amount of TTX present in *Bipalium* spp.-likely sufficient only to paralyze their prey-is extremely low (<200 ng per animal). Our estimates suggest that poisoning of mammals, including humans, would require ingestion of hundreds of worms, which is highly improbable. Consequently, accidental contact with planarians should not be considered a source of significant skin reactions or other clinical complications. Furthermore, these flatworms do not play a meaningful role in disease transmission. The primary health recommendation after inadvertent contact is simply to wash one’s hands.

## TRUTH VS. SENSATIONALISM: HOW “LIKES” TIP THE SCALE

The growing public interest and online discourse surrounding *Bipalium* spp. have paralleled a marked increase in misinformation-an Internet-driven trend with broad implications, whose causes and dynamics warrant scrutiny. Through the lenses of epistemology and modern information science, we can better understand how scientific facts are overshadowed or distorted by specific audiences, often evolving into self-reinforcing echo chambers and ideological bubbles of (dis)information^31^. Key factors examined below highlight how emotional triggers and algorithmic amplification on social media elevate unverified, sensationalist claims over evidence-based truth, thereby perpetuating this phenomenon. Despite the prevalence of viral misinformation-frequently characterized by alarmist narratives about the supposed toxicity of these planarians-publications indexed in Google Scholar consistently demonstrated a measured tone and adherence to scientific discourse.

A major explanation for the widespread dissemination of misinformation lies in its sensationalist framing and exploitation of deep-rooted psychological factors. By appealing to primal emotions, such as disgust and fear-especially toward invasive species or toxins, which trigger evolutionarily conserved responses-narratives fuel public interest, drive discourse, and perpetuate rumors^1^ ^,^ ^12^ ^,^ ^32^. Fake news headlines are often deliberately designed to be emotionally evocative, and individuals experiencing heightened emotional states (positive or negative) are more likely to believe false, but not true claims^9^ ^,^ ^32^. Even before claims about alleged poisoning risks to pets and humans emerged, sensationalist content-likely crafted to maximize engagement-had already proliferated online (Figure 2C). Hyperbolic terms such as “monster”, “megaworms”, “giant predator” and “vampire” were widely employed. Numerous alarmist assertions have distorted well-documented biological traits, such as earthworm predation and regenerative capacity (Table 1), framing hammerhead flatworms as “immortal enemies” that are indestructible and capable of limitless proliferation, akin to the Lernaean Hydra of Greek mythology. Furthermore, their phylogenetic proximity to parasitic platyhelminths (e.g., cestodes and trematodes), combined with unverified claims of ecological disruption, environmental imbalance, and agricultural damage, has been similarly sensationalized. However, no definitive scientific evidence supporting these assertions exists in the literature.

Until 2020, sensationalist content on hammerhead worms was sufficient to sustain online engagement. That year, however, a U.S. entertainment website-motivated more by clicks than by accuracy-amplified claims about toxins in *Bipalium* spp., pushing this narrative beyond academic circles. As a result, toxicity quickly became the dominant theme in non-academic publications. Over the past two years (2023-2024), the proportion of informative, evidence-based articles on Google News has declined further, while sensationalist and alarmist coverage of already debunked toxicity claims has increased (Figure 2B, 2C; Table 1). This trend underscores the link between yellow journalism, media sensationalism, and the persistence of misinformation about these species, highlighting the urgent need for ethical discussions on journalistic practices in the digital age. Notably, journalistic Notably, journalistic strategies historically associated with low-quality, niche outlets are now adopted more frequently by native online news platforms than by traditional media. On today’s digital platforms, sensationalist content drives higher social media engagement and accelerates the spread of misinformation^8^ ^,^ ^12^.

Although sensationalism has deep historical roots, its entanglement with online misinformation is relatively recent, though not without precedent. During the COVID-19 pandemic, many content creators and media outlets amplified misinformation rather than promoting accurate solutions. Debates on lockdowns, vaccines, and lethality-ranging from reasoned discussions to apocalyptic rhetoric and outright falsehoods-drove unprecedented online engagement^31^ ^,^ ^33^ ^,^ ^34^. Social media algorithms exacerbated this trend by prioritizing user attention through incessant notifications and information overload, particularly on smartphones. These platforms exploit neurobiological mechanisms such as dopamine-driven reward pathways, which, contribute to negative emotions, mental distress, and addictive behaviors, especially among adolescents^35^. This system reinforces compulsive curiosity and perpetuates cycles of sensationalism and misinformation. In this context, the relentless pursuit of engagement, at the expense of public health and scientific integrity, fuels the exploitation of sensationalist narratives. As seen during the COVID-19 pandemic, platforms prioritized click-driven content regardless of factual accuracy. Concerningly, these patterns are re-emerging in lower-stakes contexts, including the recent global rediscovery of invasive flatworm species. Such dynamics have exacerbated pseudoscientific narratives and deepened public confusion surrounding hammerhead worms.

## MEDIA AND PUBLIC ENGAGEMENT IN SCIENCE: EMERGING QUESTIONS

Hammerhead worms were likely encountered by humans centuries ago, albeit within restricted regions. Yet these organisms never achieved significant notoriety, perhaps because their perceived threat remained minimal despite their unsettling appearance. In recent years-nearly two centuries after the first scientific description of *Bipalium*-these worms have attracted widespread community and media attention, enabling a systematic assessment of their consequences (Table 1). Counterintuitively, this visibility has not necessarily produced positive outcomes and instead invites critical reflection on the role of contemporary society in science and information dissemination. In particular, it raises questions about balancing the benefits and risks of democratized online knowledge sharing-an epistemological innovation that disrupts centuries of scientific tradition.

To trace the trajectory by which *Bipalium* spp. entered broader public discourse, we analyzed key events that catalyzed this shift. The first inflection point occurred in 2017, when a viral Facebook post originating in Malaysia-initially designed to spark public curiosity-was disseminated by general science platforms and news media, thereby extending its reach to Western audiences. A second turning point followed in 2018, when media amplification intensified after the publication of a four-year survey in an open-access journal reporting new records and an updated status of invasive hammerhead flatworms in France. This study, which incorporated extensive public participation consistent with citizen science principles^29^, was widely publicized, and sensationalist terminology began to appear in coverage.

Although attempts were later made to correct the record, they remained relatively limited. By 2020, sensationalist reports regarding the toxicity of land planarians gained traction, particularly in the United States and Canada, following the aforementioned entertainment website post. What began as localized misinformation, in France expanded across North America and subsequently spread to other Western countries. In Brazil, Portuguese-language coverage was rare until 2023, when a CNN Brasil report on the situation in the U.S. triggered a surge in Portuguese Google News mentions of hammerhead worms. Although further study is warranted, these observations suggest that traditional media outlets may exert greater influence than individual influencers or internet users in disseminating information across linguistic and national boundaries. This underscores the vital role of responsible reporting by major news organizations in curbing the global spread of misinformation, as media channels govern participation in scientific discourse and shape the processes through which evidence attains credibility^10^ ^,^ ^11^. Greene^10^ emphasized that media technologies-whether print or digital-affect the production, legitimation, and dissemination of scientific knowledge, thereby shaping public engagement and perceptions of scientific facts. Many recent publications on misinformation related to *Bipalium* spp. have appeared in open science journals, whose advantages are widely discussed but which also present notable challenges^36^. As digital media increasingly disrupts traditional scientific authority, stronger media literacy and rigorous editorial standards are essential for safeguarding scientific integrity.

Although misinformation on the Internet was initially assumed to be largely orchestrated and intentional, this claim remains unconfirmed, and explanations-particularly within social network contexts-are far more complex^12^ ^,^ ^32^ ^,^ ^33^. There is no evidence that the primary misinformation discussed in this mini-review was deliberately engineered. Instead, it appears to echo ancestral behaviors driven more by emotion than reason, resembling herd behavior and linked to the pursuit of rewards such as fame and profit. These dynamics frequently escape governmental oversight, fueling ongoing debates on the regulation of social and digital media in various countries-an important issue beyond the scope of this study.

Our empirical findings on the rapid spread of misinformation about hammerhead flatworms align with established frameworks of Dynamic Social Network Modeling and Analysis, which provide a mechanistic perspective on misinformation cascades in digital environments^37^ ^,^ ^38^. These frameworks conceptualize information flow within evolving social networks, highlighting network topology, actor interactions, and feedback loops as central to information diffusion. They explicitly connect psychological factors (e.g., cognitive biases and trust dynamics) and epistemological challenges (e.g., information credibility and knowledge gaps) identified in our study to observable network phenomena such as selective exposure, clustering, and echo chambers^8^ ^,^ ^9^ ^,^ ^12^ ^,^ ^31^ ^,^ ^32^. Moreover, these models explain how behaviors that reinforce misinformation, such as homophily and confirmation bias, are amplified within tightly connected subgroups^35^ ^,^ ^37^ ^,^ ^38^, driving viral spread and entrenching false narratives. Understanding the dynamics revealed by these models-capable of both accelerating scientific knowledge dissemination and sustaining misinformation barriers-is critical for developing targeted, network-aware interventions. Such approaches are essential for designing effective science communication strategies that build public trust and foster critical engagement, particularly given the growing emphasis on public participation in scientific research^39^.

## CLOSING REMARKS AND PERSPECTIVES

It is concerning that hammerhead worms-long known to both humans and science-have recently been portrayed as highly threatening and venomous in numerous articles retrieved through Google News searches, despite the lack of supporting evidence. In such cases, media coverage and social media users amplify unverified risks, particularly false claims of extreme toxicity, which contradict both traditional knowledge and the current scientific literature. Scholarly work has instead focused on their biology and potential ecological impacts based on technical reports. This divergence illustrates broader patterns of Internet-driven misinformation and the persistence of alarmist narratives within echo chambers or epistemic bubbles, even when scientific studies provide clear counterevidence^12^ ^,^ ^31^ ^,^ ^33^.

When Justine et al.^29^ questioned how such conspicuous flatworms had escaped scientific attention for so long, they could not have foreseen that these invertebrates would later become a trending online topic, accompanied by widespread misinformation. Despite their surge in popularity, land planarians have largely escaped systematic scrutiny by scientists and authorities in many countries. This phenomenon reflects a broader challenge: while increased communication was once expected to strengthen ties between science and society, the speed, lack of quality control, and viral nature of digital information now present new obstacles. In this environment, facts can be overlooked or distorted, and once scientific topics enter mainstream discourse, they risk transforming into health misinformation crises or infodemics that deepen the gap between scientists and society.

The present case of misinformation surrounding giant hammerhead flatworms underscores the paradoxes of democratized science^39^, including the unintended consequences of widespread information dissemination. Addressing these challenges requires innovative strategies and sustained dialogue to improve online scientific communication, particularly in fields such as public health and tropical medicine. Meaningful progress will depend on authentic transdisciplinary approaches that integrate diverse perspectives to enhance public scientific understanding and counter misinformation effectively. Ensuring that individuals can access accurate, evidence-based information, comprehend it, and apply it responsibly is both feasible and essential to minimizing harm and fostering informed decision-making.

## Data Availability

Research data is only available upon request.
